# Effectiveness of mobile health in symptom management of prostate cancer patients: a systematic review and meta-analysis

**DOI:** 10.3389/fdgth.2025.1584764

**Published:** 2025-05-07

**Authors:** Hai Shan Chen, Hua He, Hai Hang Lin, Yuan Zhang, Nu Li, Ya Mei Li

**Affiliations:** Department of Urology, The Seventh Affiliated Hospital of Sun Yat-sen University, Shenzhen, China

**Keywords:** prostate cancer, mobile health (mHealth), symptom management, long-term effects, short-term effects, meta-analysis

## Abstract

**Background:**

Mobile health (mHealth) is an accessible strategy to deliver health information and is becoming increasingly popular as a form of follow-up among medical staff. However, the effects of mobile health on the physical and mental health outcomes of patients with prostate cancer after discharge from the hospital remain unclear. This meta-analysis evaluated the current evidence regarding the effects of mHealth interventions on the outcomes of patients with prostate cancer.

**Methods:**

Four databases (PubMed, Cochrane Central electronic database, EMBASE, and Web of Science) were searched from inception to 8 November 2024 for randomized controlled trials (RCTs) comparing the effects of mobile health vs. usual care on the outcomes of patients with prostate cancer. Pooled outcome measures were determined using random effects models.

**Results:**

In total, 11 RCTs, including 1,368 patients, met the criteria for inclusion in this meta-analysis. The meta-analysis revealed a significant effect of mHealth interventions on long-term bowel function outcomes (standard mean difference = 0.19, 95% confidence interval = 0.01–0.37, *P* = 0.04, I^2^ = 0.00%) compared with the usual standard care or no mHealth. However, no significant differences were observed in the following outcomes: short-term and long-term effects on anxiety, depression, self-efficacy, psychological distress, and urinary and hormonal function, and short-term effects on bowel function.

**Conclusions:**

mHealth interventions can significantly improve long-term bowel function outcomes. However, more research is needed to confirm other physical and mental health outcomes.

**Systematic Review Registration:**

https://www.crd.york.ac.uk/prospero/, PROSPERO (CRD420250651320).

## 1 Introduction

Prostate cancer (PC) is the second-most prevalent cancer in men worldwide (1). GLOBOCAN 2020 estimates reported 1,414,259 new cases of prostate cancer globally in 2020, with a higher prevalence in developed countries (2). During active treatment (prostatectomy, chemotherapy, radiotherapy, and/or hormone therapy) and the years after (survivorship), many patients experience symptom distress, including physical (urinary symptoms, bowel symptoms, sexual dysfunction, hormonal imbalance symptoms, etc.), psychological (anxiety, depression, etc.), and social aspects (stigma, social alienation, etc.), which can negatively affect quality of life (QOL) (3–5). Studies have shown that the incidence of urinary incontinence among localized prostate cancer was 4%–31%, the incidence of fecal incontinence was 4%–10%, and the incidence of sexual dysfunction was 11%–87%. These symptoms can even last as long as 10 years (6–9). Compared to men with localized disease, men with advanced prostate cancer experience greater pain and fatigue, higher levels of psychosocial distress, increased risk of suicide, and poorer health-related quality of life (10–12). Many of these symptoms persist for years, even after treatment is completed. As a result, many patients suffer from socioeconomic loss, resulting in significant life changes. Therefore, a strategy to reduce symptom distress during treatment or after discharge is of great clinical value.

Mobile health (mHealth) is an accessible strategy for delivering health information and has been widely used during the COVID-19 pandemic (13, 14). A recent meta-analysis revealed that mHealth interventions may provide an acceptable and feasible strategy to deliver continuity of health support to patients between medical appointments (15). Moreover, these interventions offer a convenient way to support self-management of cancer-related symptoms (16). Whether an individual receives treatment for PC upon diagnosis or is actively monitored over time, mHealth applications offer great opportunities for men to receive personalized, timely, high-quality, and evidence-based care (17). However, patients with prostate cancer have multiple and complex symptoms during and after treatment, with a long recovery period. After discharge, patients still need continuous and comprehensive interventions and nursing for rehabilitation training, prevention of complications, psychology, and other aspects (18). Therefore, mHealth interventions offer patients with PC an alternative form of medical care through a variety of communication technologies, including telephone, mail, remote video, or mobile applications, to treat patients at a distance.

However, the short-term and long-term effects of remote interventions on symptom management in patients with PC are unclear, and less attention has been paid to the long-term effects of the interventions. A newly published scoping review has shown that the current application of mHealth interventions in PC survivorship care pathways remains poor, with 10 mHealth apps identified and only one still in use, and the long-term effects of these apps are currently unknown (18). This research highlights the benefits of mHealth among patients with PC and shows long-term (12-month) improvements in urinary irritation, bowel function, hormonal function, and depression scores (19). However, a randomized controlled trial from Australia revealed that 12 months of telephone intervention was not accompanied by overall improvements in QOL or psychological distress (20).

A literature review found that most studies on the impact of mHealth on symptom management for patients with prostate cancer have evaluated the long-term effects of the intervention by following up patients for 3 months after the intervention (20–22). Therefore, this study focused on the short- and long-term effects of the intervention at a node of 3 months. Given these findings, it is necessary to update meta-analyses to comprehensively analyze the impact of mHealth interventions on symptom management of patients with PC.

## 2 Methods

### 2.1 Protocol registration

This meta-analysis was performed in accordance with the Preferred Reporting Items for Systematic review and Meta-Analyses (PRISMA) guidelines (23). The protocol was registered with the International Prospective Register of Systematic Reviews (PROSPERO) (Registration ID: CRD420250651320; available at: https://www.crd.york.ac.uk/prospero/).

### 2.2 Study selection

The PubMed, Cochrane Central electronic database, EMBASE, and Web of Science were searched from inception to 8 November 2024 using a series of keywords related to mobile health and prostate cancer. The details of the search strategy are presented in the Supplementary Appendix. To avoid missing any relevant studies, manual searches of the reference lists of all included articles were also performed, as were searches of the reference lists of previous related meta-analyses or systematic reviews.

The inclusion criteria for studies were as follows: (1) population: adults (age > 18 years) with a prostate cancer diagnosis who were undergoing or completed active prostate cancer treatment (surgery, chemotherapy, and/or radiotherapy); (2) intervention: mHealth interventions including but not limited to smartphone apps, SMS, or wearable devices targeting symptom monitoring or self-management; (3) control group comprised of those receiving the usual care (e.g., standard follow-up) or non-digital interventions (e.g., paper diaries); (4) primary outcomes: mental (depression, anxiety, and distress) or physical (urinary function, sexual function, bowel function, and hormonal function) health outcomes based on standardized scales with a secondary outcome of quality of life; (5) study design: randomized controlled trials (RCTs). This meta-analysis focused on the short-term and long-term effects of the intervention at the node of 3 months. The short-term effect was measured after the end of the intervention, and the measured outcome indicators were followed up for ≤3 months. The long-term effect was measured after the end of the intervention, and the measured outcome indicators were followed up for >3 months (we selected the longest time point as the data outcome).

Studies were excluded from this meta-analysis according to the following exclusion criteria: (1) publication in any language other than English or (2) presentation of incomplete data or duplicated data.

EndNote X9 (Clarivate Analytics, https://www.endnote.com) was used to manage references. Two authors (HSC and HH) independently examined titles and abstracts to select eligible trials. In cases of discrepancies between the two authors' selections, a third author (YML) was consulted to resolve the differences. Full-text articles were then retrieved and reviewed using the same method to determine which trials met the inclusion criteria for this study.

### 2.3 Data extraction

Two authors (HH and HHL) independently extracted data from the included trials using a data extraction table that included general information (title, first author, publication date, and study location), trial characteristics (sample size, method of randomization, allocation, blinding method, incomplete outcomes data, selective reporting, and other), subject characteristics (country, neoplasm staging, treatment condition, and age), intervention (type of intervention, intervention and follow-up durations, frequency of mHealth use, intervener, and theoretical framework), and outcomes (outcome measures). Discrepancies between authors were resolved through consultation with a third author (HSC).

### 2.4 Risk of bias assessment

In this study, two reviewers (HSC and HH) assessed the quality of the included studies using the Risk of Bias 2 (RoB 2) tool, as recommended by the Cochrane Handbook (24). This tool evaluates the risk of bias in five domains: bias arising from the randomization process, bias due to deviations from intended interventions, bias due to missing outcome data, bias in the measurement of the outcome, and bias in the selection of the reported result. An overall risk of bias assessment was also conducted for each study. Disagreements with respect to the methodological quality were resolved by discussion and mutual consultation (YML).

### 2.5 Statistical analysis

The effects of mHealth on the outcomes of patients with PC were evaluated on the basis of the included studies. Continuous variables were evaluated and combined using mean difference (MD) when units of measurement were consistent across studies or could be converted to a common scale. When the units could not be directly compared or converted, the standardized mean difference (SMD) was used (24). The statistical parameter I^2^ was used to examine the heterogeneity of the effect sizes, with values higher than 50% indicating substantial heterogeneity (24). In this study, considering the existence of clinical heterogeneity (e.g., mode of intervention, participant characteristics), we employed the DerSimonian and Laird random effects model for our meta-analysis (24). A sensitivity analysis was conducted by removing the RCTs that included interventions that relied entirely on mHealth, those that included interventions that were delivered by peer support volunteers, or those that included a theory or framework referenced across the studies to detect the stability of the results. If the number of included studies for the outcome indicators was more than 10, a funnel plot and Egger’s test were used to detect publication bias. The level of statistical significance was set at *P* < 0.05 and all statistical tests were two-sided. Statistical analyses were performed using STATA version 17.0 (Stata Corp LP, College Station, TX, USA).

## 3 Results

### 3.1 Study identification and selection

**Figure 1:**
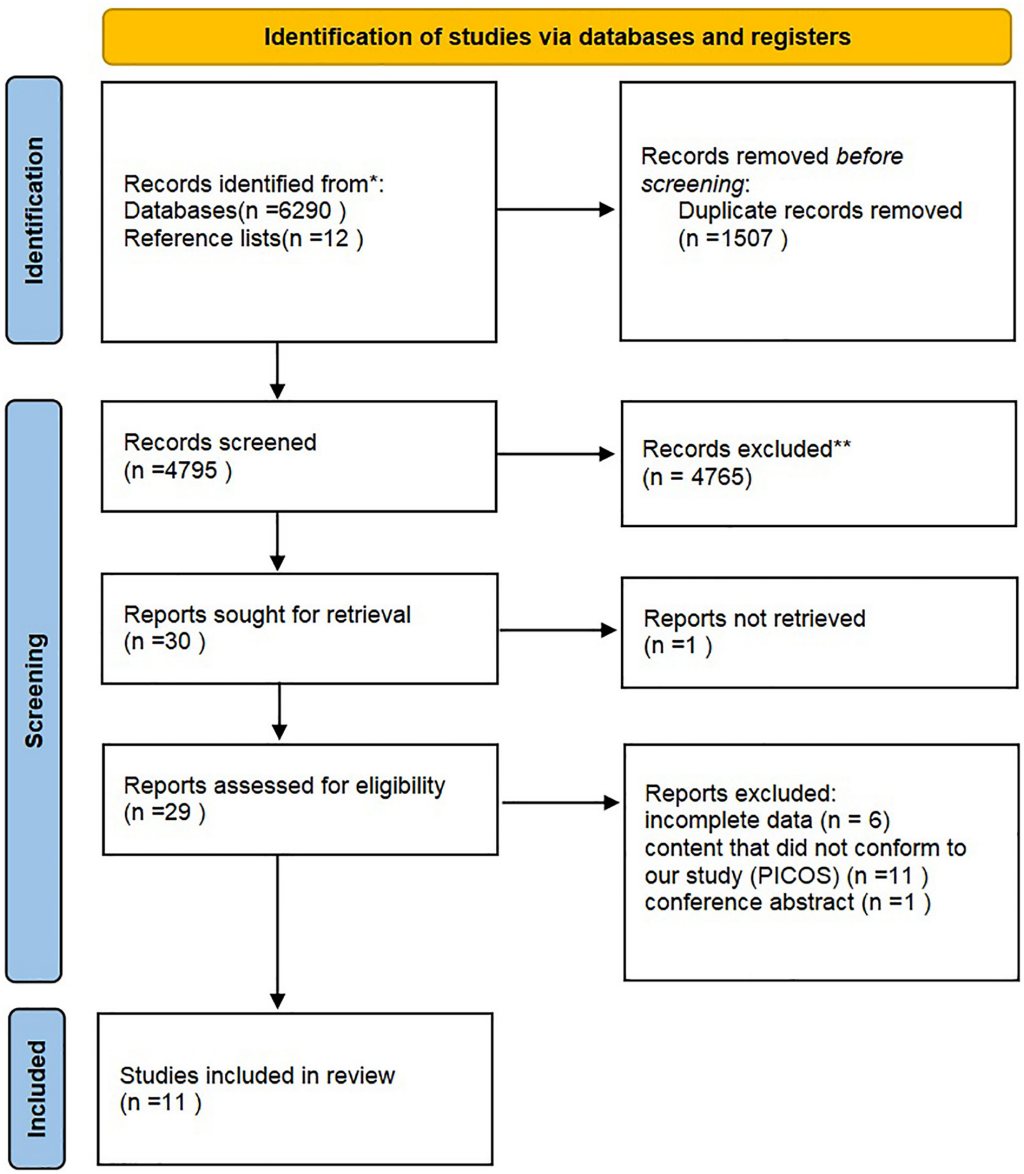
Inclusion screening flow chart.

The initial search identified 6,302 articles. After screening of titles and abstracts, 6,273 articles were excluded, including 1,507 duplicates, and 4,766 articles that did not meet the inclusion criteria. The full texts of 29 articles were reviewed, and 18 of these articles were excluded due to incomplete data (*N* = 6) (25–29), content that did not conform to our study (PICOS) (*N* = 11) (30–40), or being a conference abstract (*N* = 1) (37). Finally, 11 RCTs were included in the present meta-analysis (Figure 1).

The characteristics of the included RCTs are summarized in Table 1. The 11 RCTs included two studies (18%) conducted in Australia, Canada, the UK, the USA, and Korea, and one (9%) study conducted in Germany. All RCTs were published in English and conducted between 2013 and 2022. A total of 1,368 patients diagnosed with prostate cancer were included. Among the 11 included RCTs, the study of Galvão et al. (20) had the largest sample size with 463 individuals. Except for two RCTs, which used an Internet intervention, all other mHealth interventions were performed over the telephone. Interventions ranged from 6 weeks to 6 months in duration, and the studies were delivered by professional technical personnel (including clinical psychologists, clinicians, physicians, clinical nutritionists, nurses, and research assistants) (*n* = 10) and peer support volunteers (*n* = 1). Most interventions were delivered by health and social care professionals, and only one relied entirely on mHealth. One theory or framework was referenced across studies (21). The control group received the usual care, ethical care, guidelines for patients with prostate cancer, or health promotion.

**Table 1:** Characteristics of the included articles.

Study	Country	Design	Neoplasm staging	Treatment condition	No. of participants		Mean age ± SD		Type of intervention		Theory or framework	Outcome
							(years)					
					Trial control		Trial control		Trial control			
Davis et al. (41)	USA	RCT, two arms	Early-stage PC survivors	RP, RT, ADT, watchful waiting	49	45	61.9 ± 7.0	62.0 ± 8.1	Form: telephone	Usual care	NA	General health-related quality of life (HRQOL), cancer-specific HRQOL, prostate cancer–specific HRQOL, Post-visit ratings (PVR)
									Content: symptom monitoring plus feedback			
									Frequency: —			
									Time: three telephone interviews			
									Intervenor: research assistant (RA)			
Hunter et al. (42)	UK	RCT, two arms	Localized (50%), locally advanced (19%), and metastatic cancer (31%)	ADT	33	35	67.97 ± 7.65	69.71 ± 7.9	Form: a booklet, CD, and telephone contact	Usual care	NA	Hot flushes and night sweats (HFNS)-Hot Flush Rating Scale
												(HFRS), Hospital Anxiety and Depression Scale (HADS), health-related quality of life
									Content: guided self-help cognitive behavioural therapy (CBT)			
									Frequency: —			
									Time: 6 weeks			
									Intervenor: clinical psychologist (ES)			
Chambers et al. (22)	Australia	RCT, three arms	Localized prostate cancer	RP	61	64	NA	NA	Form: telephone	Usual care	NA	Sexual function, sexuality needs, sexual self-confidence, masculine self-esteem, marital satisfaction or intimacy
									Content: nurse counseling			
									Frequency: —			
									Time: —			
									Intervenor: prostate cancer nurse			
					64	64	NA	NA	Form: telephone	Usual care	NA	
									Content: peer support			
									Frequency: —			
									Time: —			
									Intervenor: peer support volunteers (Peers)			
Lambert et al. (43)	Canada	RCT, two arms	Diagnosed in the past 4 months	RP, RT, ADT, watchful waiting or active surveillance	23	19	64.3 ± 7.7	63.1 ± 5.6	Form: telephone	Minimal ethical care (MEC)	NA	Anxiety (HADS-A), depression (HADS-D), self-efficacy, quality of life, relationship satisfaction, dyadic coping (DCI), Illness appraisal, individual coping (Brief COPE)
									Content: coping together (CT), i.e., self-directed coping skill intervention for couples facing cancer			
									Frequency: fortnightly			
									Time: 2 months			
									Intervenor: clinicians			
Lange et al. (44)	Germany	RCT, 2 arms	—	Prostatectomy	18	26	60.53 ± 6.70	62.77 ± 6.10	Form: telephone	German S3 guideline for prostate cancer patients	NA	Distress, anxiety, depression, anger, need for help, QOL, fear of progression (FoP) and coping with cancer
									Content: five group sessions in three different chat groups			
									Frequency: weekly			
									Time: each session lasted 60–90 min			
									Intervenor: psychologist			
Galvão et al. (20)	Australia	RCT, two arms	Localized prostate cancer	Have undergone or are currently undergoing PC treatment	232	231	NA	NA	Form: telephone, web	Usual care	NA	Health-related QOL, disease-specific QOL, EPIC
									Content: peer-led intervention, self-management materials, and monthly telephone-based group peer support			
									Frequency: monthly			
									Time: 6 months			
									Intervenor: peer, PC nurse counselor			
McCaughan et al. (46)	UK	RCT, two arms	Localized prostate cancer	Post-surgical or post-radiotherapy treatment with or without hormone treatment	26	8	NA	NA	Form: telephone	Usual care	NA	Self-efficacy; quality of life, symptom distress, communication, uncertainty and illness benefits
									Content: five intervention sessions			
									Frequency: —			
									Time: 9 weeks			
									Intervenor: facilitator-led			
Saengryeol et al. (45)	Korea	RCT, two arms	Advanced PC	ADT	11	10	66.0 (61.0–71.0)	67.0 (59.5–73.0)	Form: motivational text messages	Usual care	NA	QOL, life satisfaction, anxiety and depression
									Content: structured lifestyle intervention			
									Frequency: 2 supervision visits (week 4 and week 8) and one main tenance visit (week 12)			
									Time: 3–8 weeks			
									Intervenor: —			
Park et al. (47)	Korea	RCT, two arms	—	ADT	86	86	66.3, 65.0 (±6.8)	66.5, 66.0 (±8.2)	Form: Android-based smartphone, a smartphone application, a web-based platform, and a smartband (Neofit band, KT, Korea)	Usual care	NA	Vital sign measurements, physical measurements, cardiorespiratory endurance, physical strength, self-reported physical activity, QOL
									Content: The Smart After-Care (SAC) service			
									Frequency: weekly			
									Time: 12 weeks			
									Intervenor: physicians, clinical nutritionists, and exercise therapists			
Benzo et al. (19)	USA	RCT, two arms	Stage-III or IV prostate cancer	ADT	95	97	NA	NA	Form: web	Health promotion (HP)	NA	Five domains of symptom-related quality of life
									Content: cognitive-behavioral stress management (CBSM)			
									Frequency: weekly			
									Time: 10 weeks			
									Intervenor: clinical psychologists			
Lambert et al. (21)	Canada	RCT, two arms	Prostate cancer (local or metastasized)	Surgery, chemotherapy, radiation therapy, hormone therapy, and/or brachytherapy	26	23	≤60: 6 (23.1), ≥61: 20 (76.9)	≤60: 6 (26.1), ≥61: 17 (73.9)	Forms: web	Usual care	Stress and coping framework, framework of dyadic coping, self-efficacy theory	Anxiety, QOL, depression, self-management skills, physical activity, self-efficacy, appraisal
									Content: TEMPO—the first dyadic, tailored, web-based, psychosocial and physical activity self-Management PrOgram feedback			
									Frequency: —			
									Time: 10 weeks			
									Intervenor: RAs			

RP, radical prostatectomy; RT, radiation therapy, ADT, Androgen deprivation therapy, CD, Compact disc.

### 3.2 Risk of bias assessment

**Figure 2:**
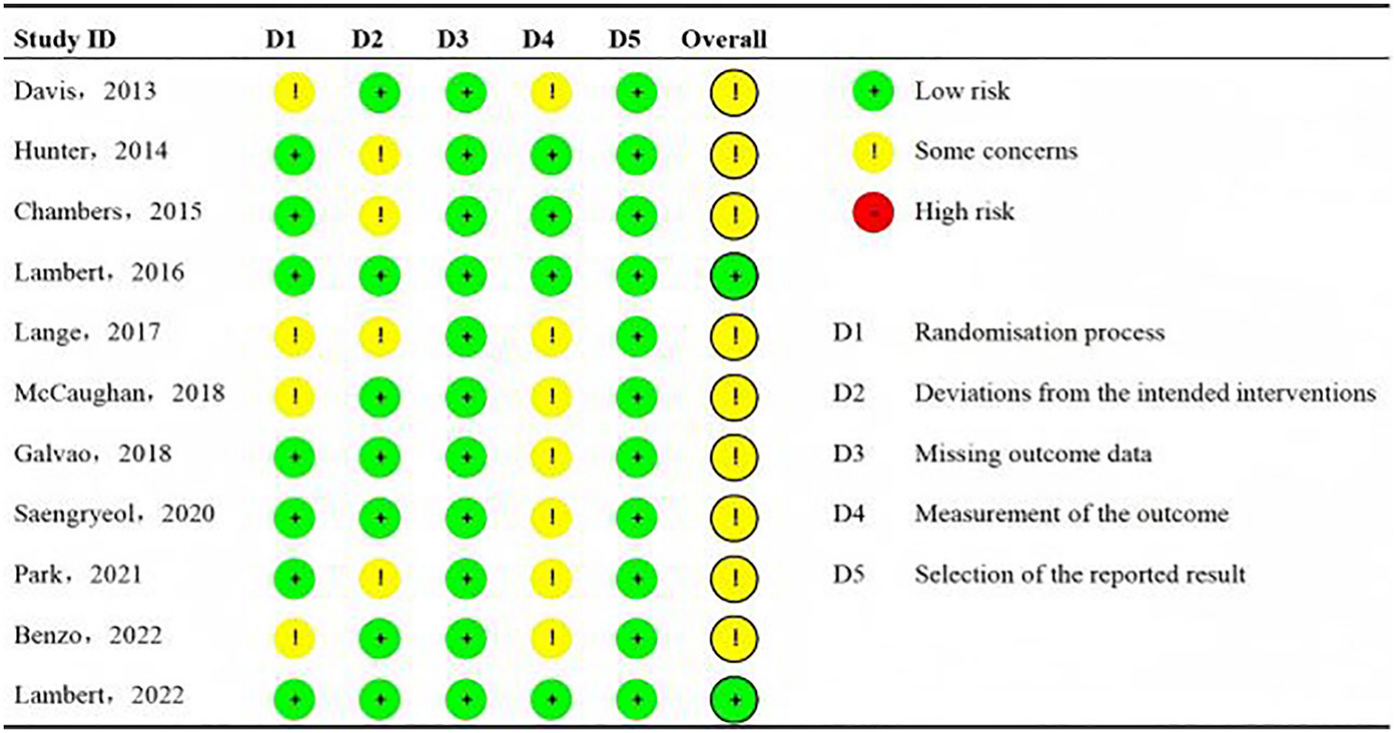
Risk of bias summary.

The Cochrane Collaboration RoB tool was used to assess the RoB of the included studies. The domains for assessment included selection bias, including sequence generation and allocation sequence concealment; performance or detection bias via blinding of participants, personnel, and outcome assessors; attrition bias via incomplete outcome data; and reporting bias via selective outcome reporting. The criteria for low, unclear, and high RoB within and across the studies followed the Cochrane Handbook for Systematic Reviews of Interventions. RoB was independently assessed by authors HSC (five articles), HH (three articles), and HHL (three articles). YML reviewed all RoB assessments to confirm accuracy. In addition, data integrity and selective reporting were both assessed as low risk. The results are presented in Figure 2.

### 3.3 Meta-analysis of primary outcomes

Six RCTs (20, 21, 42–45) (*n* = 687 participants) reported the short-term effect of the intervention on anxiety, and the meta-analysis demonstrated no significant difference between the mHealth intervention group and the control group [SMD = −0.07, 95% confidence interval (CI) = −0.43 to 0.29, *P* = 0.70, I^2^ = 63.30%] (Table 2). The long-term effects of the interventions on anxiety were measured in two studies (*n* = 419 participants) (20, 42), and the meta-analysis demonstrated no significant difference between the mHealth intervention group and the control group (SMD = −0.11, 95% CI = −0.30 to 0.08, *P* = 0.26, I^2^ = 0.00%) (Table 3).

**Table 2:** The short-term effect of mHealth interventions on anxiety, depression, self-efficacy, psychological distress, urinary function, and bowel function outcomes from randomized controlled trials.

Outcome	Studies	No. of participants		Heterogeneity test I^2^(%)	Effect size			
		Trial	Control		SMD	95% CI		*P*
Anxiety	6	283	306	63.30	−0.07	−0.43	0.29	0.70
Depression	6	283	306	54.23	−0.08	−0.40	0.24	0.63
Self-efficacy	2	31	15	0.00	−0.02	−0.84	0.44	0.54
Psychological distress	4	70	64	45.39	−0.01	−0.52	0.50	0.96
Urinary function	2	259	280	54.45	0.22	−0.05	0.05	0.12
Bowel function	2	259	280	71.41	0.21	−0.14	0.57	0.24

The short-term effect was measured after the end of the intervention, and the measured outcome indicators were followed up for ≤3 months.

**Table 3:** The long-term effect of mHealth interventions on anxiety, depression, urinary function, bowel function, hormonal function and sexual function outcomes from randomized controlled trials.

Outcome	Studies	No. of participants		Heterogeneity test I2(%)	Effect size			
		Trial	Control		SMD	95% CI		*P*
Anxiety	2	208	211	0.00	−0.11	−0.30	0.08	0.26
Depression	3	221	225	26.09	−0.18	−0.49	0.13	0.25
Urinary function	3	255	274	0.00	0.06	−0.11	0.23	0.51
Bowel function	3	228	245	0.00	0.19	0.01	0.37	0.04
Hormonal function	2	245	267	0.00	0.11	−0.06	0.28	0.21
Sexual function	4	389	348	93.82	−0.05	−0.67	0.56	0.86

The long-term effect was measured after the end of the intervention, and the measured outcome indicators were followed up for >3 months.

Six RCTs (20, 21, 42–45) (*n* = 589 participants) reported the short-term effect of the intervention on depression, and the meta-analysis demonstrated no significant difference between the mHealth intervention group and the control group (SMD = −0.08, 95% CI = −0.40 to 0.24, *P* = 0.63, I^2^ = 54.23%) (Table 2). The long-term effects of the interventions on depression were measured in three studies (19, 20, 42) (*n* = 446 participants), and the meta-analysis demonstrated no significant difference between the mHealth intervention group and the control group (SMD = −0.18, 95% CI = −0.49 to 0.13, *P* = 0.25, I^2^ = 26.09%) (Table 3).

Two RCTs (43, 46) (*n* = 46 participants) reported the short-term effect of the interventions on self-efficacy, and the meta-analysis demonstrated no significant difference between the mHealth intervention group and the control group (SMD = −0.02, 95% CI = −0.84 to 0.44, *P* = 0.54, I^2^ = 0.00%) (Table 2).

Four RCTs (21, 43, 44, 46) (*n* = 134 participants) reported the short-term effect of the interventions on psychological distress, and the meta-analysis demonstrated no significant difference between the mHealth intervention group and the control group (SMD = −0.01, 95% CI = −0.52 to 0.50, *P* = 0.96, I^2^ = 45.39%) (Table 2).

Two RCTs (20, 47) (*n* = 539 participants) reported the short-term effect of the interventions on urinary function, and the meta-analysis demonstrated no significant difference between the mHealth intervention group and the control group (SMD = 0.22, 95% CI = −0.05 to 0.50, *P* = 0.12, I^2^ = 54.45%) (Table 2). The long-term effects of the interventions on urinary function were measured in three studies (19, 20, 41) (*n* = 529 participants), and the meta-analysis demonstrated no significant difference between the mHealth intervention group and the control group (SMD = 0.06, 95% CI = −0.11 to 0.23, *P* = 0.51, I^2^ = 0.00%) (Table 3).

**Figure 3:**
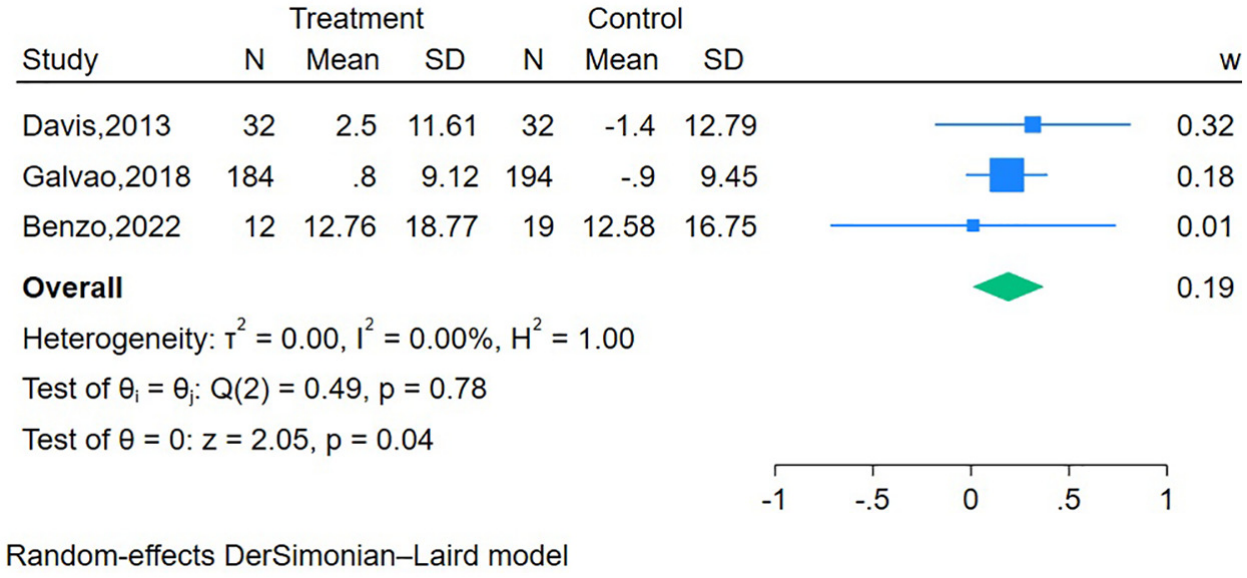
Forest plot of the long-term effect of mHealth interventions on bowel function.

Two RCTs (20, 47) (*n* = 539 participants) reported the short-term effect of the interventions on bowel function, and the meta-analysis demonstrated no significant difference between the mHealth intervention group and the control group (SMD = 0.21, 95% CI = −0.14 to 0.57, *P* = 0.24, I^2^ = 71.41%) (Table 2). The long-term effects of the interventions on bowel function were measured in three studies (19, 20, 41) (*n* = 473 participants), and the meta-analysis demonstrated a significant difference between the mHealth intervention group and the control group (SMD = 0.19, 95% CI = 0.01–0.37, *P* = 0.04, I^2^ = 0.00%) (Figure 3).

The long-term effects of the interventions on hormonal function were measured in two studies (19, 20) (*n* = 512 participants), and the meta-analysis demonstrated no significant difference between the mHealth intervention group and the control group (SMD = 0.11, 95% CI = −0.06 to 0.28, *P* = 0.21, I^2^ = 0.00%) (Table 3).

The long-term effects of the interventions on sexual function were measured in four studies (19, 20, 22, 41) (*n* = 737 participants), and the meta-analysis demonstrated no significant difference between the mHealth intervention group and the control group (SMD = −0.05, 95% CI = −0.67to 0.56, *P* = 0.86, I^2^ = 93.82%) (Table 3).

### 3.4 Meta-analysis of secondary outcomes

As for the secondary outcomes (Table 4), the meta-analysis demonstrated no significant differences in the short-term effects of the interventions on quality of life, mental health, and physical health. Three health-related QOL (HRQOL) measures, validated in patients with prostate cancer, were used: the physical and mental scales of the Assessment of Quality of Life-8 Dimensions (AQoL-8D) (43), the 8-item Short Form Health Survey (SF-8) (44), and the 12-item Short Form Health Survey (SF-12) (21).

**Table 4:** Meta-analysis of secondary outcomes.

Outcome	Studies	No. of participants		Heterogeneity test I^2^(%)	Effect size			
		Trial	Control		SMD	95% CI		*P*
Quality of life	3	207	219	43.58	0.10	−0.44	0.64	0.71
Mental health	3	58	62	0.00	−0.11	−0.47	0.25	0.56
Physical health	3	58	62	0.00	−0.13	−0.49	0.23	0.48

The short-term effect of mHealth interventions on quality of life, mental health, and physical health outcomes from randomized controlled trials.

**Figure 4:**
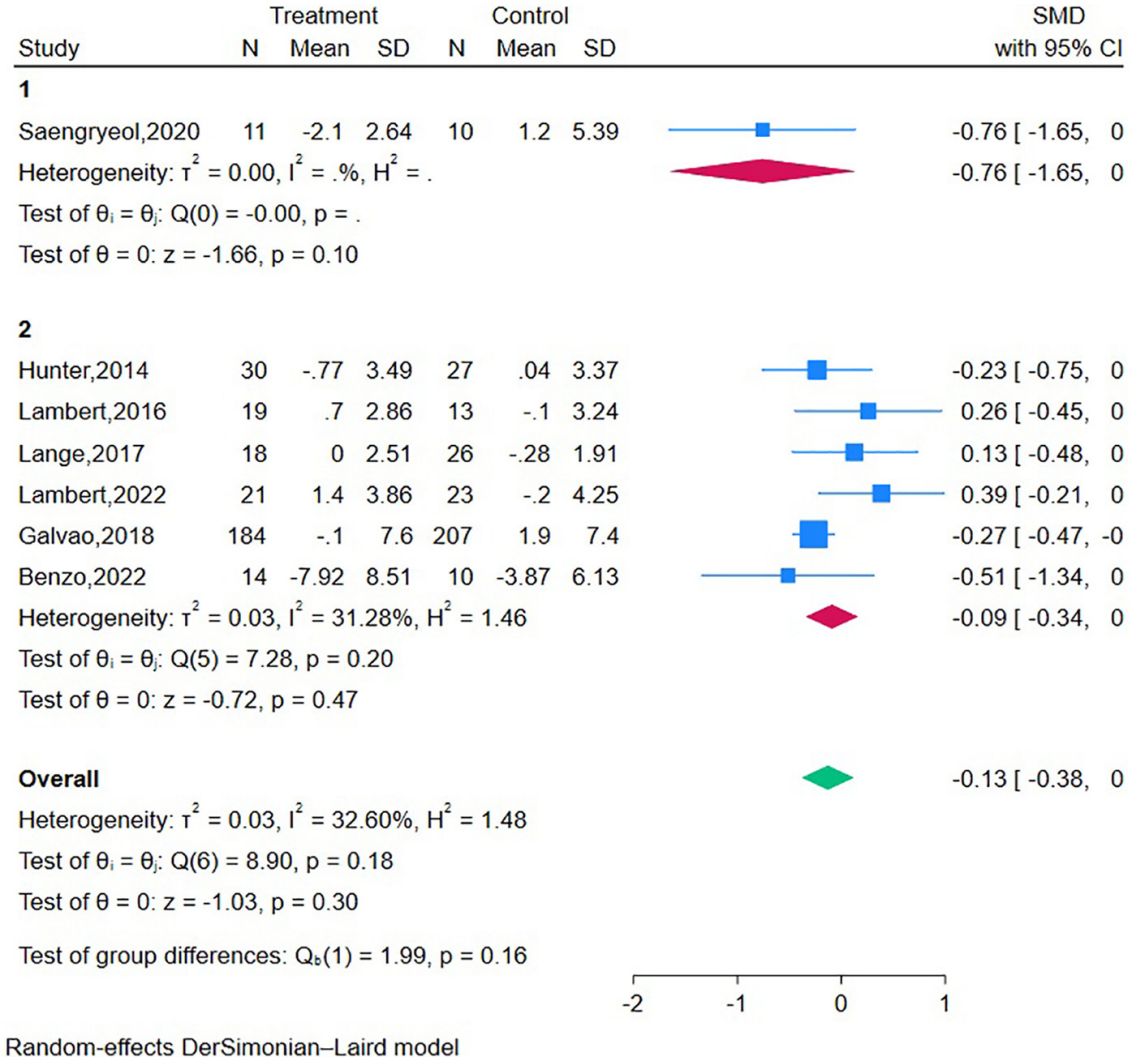
Subgroup analysis.

Three RCTs (19, 20, 41) reported that the mHealth interventions were delivered by professional technical personnel, and one RCT (22) reported that the mHealth intervention was delivered by peer support volunteers. In the sensitivity analyses (Table 5), after removing the RCT of Chambers et al. (22), the sexual function differences became statistically significant. However, since only three studies were included, further research is needed to explore these findings. Subgroup analysis was conducted on data of tumor stage and intervention type (Figure 4). Relevant hypothesis tests indicated no significant differences.

**Table 5:** Sensitivity analysis of intervention heterogeneity.

	All studies	Omitted Chambers (22)	Omitted Saengryeol (45)	Omitted Lambert (21)
Sexual function	−0.05 (−0.67 to 0.56)	0.24 (0.06 to 0.43)	—	—
SMD (95% CI)				
Depression	−0.08 (−0.40 to 0.24)	—	0.11 (−0.31 to 0.10)	−0.17 (−0.49 to 0.15)
SMD (95% CI)				
Anxiety	−0.07 (−0.43 to 0.28)	—	−0.09 (−0.26 to 0.07)	—
SMD (95% CI)				
Quality of life	0.10 (−0.44 to 0.64)	—	0.02 (−0.17 to 0.22)	−0.01 (−0.47 to 0.44)
SMD (95% CI)				
Psychological distress	−0.01 (−0.52 to 0.50)	—	—	−0.05 (−0.81 to 0.70)
SMD (95% CI)				

SMD, standardized mean difference. — indicates that the trial was not included in this outcome and a sensitivity analysis could not be performed.

Studies with peer support volunteers or interventions that relied entirely on mHealth were excluded.

### 3.5 Publication bias

Fewer than 10 articles were included for each outcome, and thus, an assessment of publication bias could not be conducted.

## 4 Discussion

This meta-analysis included 11 RCTs involving 1,368 participants and provided evidence that mHealth interventions significantly improve long-term bowel function outcomes. Moreover, in the sensitivity analyses, the sexual function differences became statistically significant. However, mHealth interventions did not significantly reduce symptom distress (anxiety, depression, self-efficacy, psychological distress, urinary function, bowel function, and hormonal function outcomes) or improve quality of life.

Our findings are consistent with previous systematic literature reviews (48), which concluded that technology-based interventions (TBIs) cannot improve health outcomes (anxiety, depression, and HRQOL) among patients with prostate cancer. However, the present meta-analysis included more patient outcomes, such as self-efficacy, psychological distress, urinary function, bowel function, and hormonal function, to further explore the short-term and long-term effects of mHealth interventions.

The mHealth interventions did not improve anxiety, depression, or psychological distress symptoms. This result may be due to a floor effect, whereby participants' baseline anxiety, depression, and psychological distress symptom scores were within the healthy range. The incidence of anxiety, depression, and psychological distress among patients with prostate cancer ranges from 15% to 22% (49, 50), 14.7% to 65.9% (51, 52), and 21% to 28% (53, 54), respectively. The studies in this review did not recruit patients with anxiety or depression. In primary care, some evidence suggests that mHealth interventions can decrease anxiety, depression, and psychological distress symptoms, and there is growing evidence of their benefits in cancer care (16, 55). However, more research is needed to evaluate their effectiveness in patients with prostate cancer experiencing anxiety, depression, and psychological distress.

The present study did not find that mHealth interventions can significantly improve patients' urinary, bowel, and hormonal function outcomes, probably due to the small number of included studies (only two or three RCTs) or the fact that the included population did not comprise patients with advanced prostate cancer. Moreover, the content and frequency of mHealth interventions may not have been standardized across studies, as latest RCTs indicate that targeting a web-based intervention to recipients most likely to benefit patients with elevated levels of symptom burden and can improve urinary irritation, bowel function, hormonal function, and depression symptoms in men with PC (19). For the symptoms mentioned above, data from the studies incorporated were inconsistent, making conclusions regarding their management through mHealth interventions difficult to draw. However, the long-term effects of mHealth interventions on bowel function had a significant positive impact. Several hypotheses could explain why we observed improvements over time in these conditions. First, we hypothesize that the “attention” provided to participants (60–90 min/session in the intervention group and 0–60 min/session in the control group) by the study staff could have played a role in the improvement of symptoms over time in these conditions. Second, participation bias (e.g., participants' desire to reduce their stress) may also partially explain why individuals in these conditions improved their outcomes. Finally, we also hypothesize that improvement in bowel function across conditions could be attributed to the overlapping content presented (e.g., education component, attention, and social support). This is consistent with previous work that showed mHealth interventions as a possible method for reducing the severity of participants' irritable bowel syndrome symptoms (56), and a review that concluded that mHealth interventions were associated with significant reductions in bowel symptoms and improvement in quality of life that tended to continue up to 12 months of follow-up (57). More research is needed to elucidate and disentangle the effects of mHealth interventions on symptom-related quality of life among prostate cancer survivors.

Quality of life is related to physical health, psychological conditions, social relationships, and environment (58). mHealth interventions likely cannot improve quality of life and self-efficacy because mHealth interventions fail to improve the symptom-related distress and psychological distress of patients with prostate cancer. This result may be due to the quality of life and the self-efficacy of these men already being at normative levels at baseline, and they may not have needed an intervention. However, a recent review found that mHealth interventions are promising for improving the QOL of patients with cancer, and the strongest evidence exists for physical activity interventions, followed by mindfulness and cognitive behavioral therapy (59). This meta-analysis only included two articles on physical activity interventions and one article on cognitive behavioral therapy interventions, thus, robust conclusions cannot be drawn from the available evidence.

The latest studies show that cancer survivors benefit variably from mHealth tools (15, 16, 60). To maximize the effects of such tools, future research should consider the following aspects. First, positive associations have primarily been demonstrated between mHealth/digital literacy levels and the utilization of the Internet as part of information-seeking related to healthcare and prostate cancer; however, PC survivors' mHealth literacy levels were likely to be at a novice level. Therefore, it is necessary for us to reliably measure mHealth literacy, improve our ability to identify low-literacy target groups, and develop and test interventions to effect change (61). Second, another research study found that mHealth intervention personalization improves engagement and efficacy (62). Future studies should consider using the technology acceptance model to codesign mHealth interventions with end-users and analyze end-user personalization regarding engagement and health outcomes (15). Third, a retrospective study demonstrated that utilizing mobile Internet management for the ongoing care of patients who have undergone radical prostatectomy yields significant benefits because the invasive nature of radical prostatectomy often induces psychological stress (63). Future research could start with the treatment methods adopted by patients and analyze whether different treatment approaches have an impact on the physical and mental outcomes of patients. Fourth, future mobile health interventions should be based on patients' unique needs and preferences, considering critical factors for implementing mHealth interventions, including perceived utility, ease of use, and resolving technical barriers such as privacy, cost, and security, as well as the remote monitoring of patients' progress. Importantly, intervention measures designed jointly with end-users can enhance participation.

The present meta-analysis had several limitations. First, due to the limited number of included articles, we could not fully evaluate publication bias, which resulted in low statistical efficiency. Second, most interventions were delivered over a relatively short period, typically ranging from 6 to 10 weeks; most frequently, the interventions were provided weekly. However, the lack of data prevented the comparison of mHealth interventions by length or frequency of the intervention. In the same way, evidence was insufficient to enable comparison by neoplasm staging and perform subgroup analyses, which would have been useful because an RCT suggests that different forms of interventions may be more or less effective according to disease stage (64). Finally, different instruments were used to assess patient outcomes, which made a comparison of the results difficult.

## 5 Conclusions

The results of this systematic review and meta-analysis indicate that patients with prostate cancer benefit less from mHealth interventions both mentally (anxiety, depression, self-efficacy, psychological distress, and quality of life) and physically (urinary function and hormonal function). However, mHealth interventions significantly improved long-term bowel function outcomes. Further, we need studies with sufficient statistical power and length of follow-up to generate much-needed definitive evidence concerning the efficacy of mHealth interventions for the management of symptoms in patients with prostate cancer.

## Data Availability

The original contributions presented in the study are included in the article/Supplementary Material, further inquiries can be directed to the corresponding authors.

## References

[1] Brausi M Hoskin P Andritsch E Banks I Beishon M Boyle H ECCO essential requirements for quality cancer care: prostate cancer. Crit Rev Oncol Hematol. (2020) 148:102861. 10.1016/j.critrevonc.2019.102861 32151466

[2] Sung H Ferlay J Siegel RL Laversanne M Soerjomataram I Jemal A Global cancer statistics 2020: GLOBOCAN estimates of incidence and mortality worldwide for 36 cancers in 185 countries. CA Cancer J Clin. (2021) 71(3):209–49. 10.3322/caac.21660 33538338

[3] Mundle R Afenya E Agarwal N. The effectiveness of psychological intervention for depression, anxiety, and distress in prostate cancer: a systematic review of literature. Prostate Cancer Prostatic Dis. (2021) 24(3):674–87. 10.1038/s41391-021-00342-3 33750905

[4] Spratt DE Shore N Sartor O Rathkopf D Olivier K. Treating the patient and not just the cancer: therapeutic burden in prostate cancer. Prostate Cancer Prostatic Dis. (2021) 24(3):647–61. 10.1038/s41391-021-00328-1 33603236 PMC8384628

[5] Kazlauskas E Patasius A Kvedaraite M Nomeikaite A Rudyte M Smailyte G. Icd-11 adjustment disorder following diagnostic procedures of prostate cancer: a 12-month follow-up study. J Psychosom Res. (2023) 168:111214. 10.1016/j.jpsychores.2023.111214 36905705

[6] Al Awamlh BA Wallis CJD Penson DF Huang LC Zhao Z Conwill R Functional outcomes after localized prostate cancer treatment. Jama. (2024) 331(4):302–17. 10.1001/jama.2023.26491 38261043 PMC10807259

[7] Wallis CJD Glaser A Hu JC Huland H Lawrentschuk N Moon D Survival and complications following surgery and radiation for localized prostate cancer: an international collaborative review. Eur Urol. (2018) 73(1):11–20. 10.1016/j.eururo.2017.05.055 28610779

[8] Nam RK Cheung P Herschorn S Saskin R Su J Klotz LH Incidence of complications other than urinary incontinence or erectile dysfunction after radical prostatectomy or radiotherapy for prostate cancer: a population-based cohort study. Lancet Oncol. (2014) 15(2):223–31. 10.1016/s1470-2045(13)70606-5 24440474

[9] Ficarra V Novara G Rosen RC Artibani W Carroll PR Costello A Systematic review and meta-analysis of studies reporting urinary continence recovery after robot-assisted radical prostatectomy. Eur Urol. (2012) 62(3):405–17. 10.1016/j.eururo.2012.05.045 22749852

[10] Holm M Doveson S Lindqvist O Wennman-Larsen A Fransson P. Quality of life in men with metastatic prostate cancer in their final years before death—a retrospective analysis of prospective data. BMC Palliat Care. (2018) 17(1):126. 10.1186/s12904-018-0381-6 30509249 PMC6278096

[11] Noriega Esquives B Lee TK Moreno PI Fox RS Yanez B Miller GE Symptom burden profiles in men with advanced prostate cancer undergoing androgen deprivation therapy. J Behav Med. (2022) 45(3):366–77. 10.1007/s10865-022-00288-4 35107655 PMC9167233

[12] Crump C Stattin P Brooks JD Sundquist J Bill-Axelson A Edwards AC Long-term risks of depression and suicide among men with prostate cancer: a national cohort study. Eur Urol. (2023) 84(3):263–72. 10.1016/j.eururo.2023.04.026 37169640 PMC10523908

[13] Tosoni S Voruganti I Lajkosz K Mustafa S Phillips A Kim SJ Patient consent preferences on sharing personal health information during the COVID-19 pandemic: “the more informed we are, the more likely we are to help”. BMC Med Ethics. (2022) 23(1):53. 10.1186/s12910-022-00790-z 35596210 PMC9122733

[14] Rittberg R Mann A Desautels D Earle CC Navaratnam S Pitz M. Canadian cancer centre response to COVID-19 pandemic: a national and provincial response. Curr Oncol. (2020) 28(1):233–51. 10.3390/curroncol28010026 33704191 PMC7900889

[15] Singleton AC Raeside R Hyun KK Partridge SR Di Tanna GL Hafiz N Electronic health interventions for patients with breast cancer: systematic review and meta-analyses. J Clin Oncol. (2022) 40(20):2257–70. 10.1200/jco.21.01171 35500200 PMC9273371

[16] Ream E Hughes AE Cox A Skarparis K Richardson A Pedersen VH Telephone interventions for symptom management in adults with cancer. Cochrane Database Syst Rev. (2020) 6(6):Cd007568. 10.1002/14651858.CD007568.pub2 32483832 PMC7264015

[17] Jamnadass E Rai BP Veneziano D Tokas T Rivas JG Cacciamani G Do prostate cancer-related mobile phone apps have a role in contemporary prostate cancer management? A systematic review by Eau young academic urologists (Yau) urotechnology group. World J Urol. (2020) 38(10):2411–31. 10.1007/s00345-020-03197-w 32322996 PMC7508932

[18] Ogunsanya ME Sifat M Bamidele OO Ezenwankwo EF Clifton S Ton C Mobile health (Mhealth) interventions in prostate cancer survivorship: a scoping review. J Cancer Surviv. (2023) 17(3):557–68. 10.1007/s11764-022-01328-3 36627464 PMC13014381

[19] Benzo RM Moreno PI Noriega-Esquives B Otto AK Penedo FJ. Who benefits from an Ehealth-based stress management intervention in advanced prostate cancer? Results from a randomized controlled trial. Psychooncology. (2022) 31(12):2063–73. 10.1002/pon.6000 35851976 PMC10472415

[20] Galvão DA Newton RU Girgis A Lepore SJ Stiller A Mihalopoulos C Randomized controlled trial of a peer led multimodal intervention for men with prostate cancer to increase exercise participation. Psychooncology. (2018) 27(1):199–207. 10.1002/pon.4495 28685892

[21] Lambert SD Duncan LR Culos-Reed SN Hallward L Higano CS Loban E Feasibility, acceptability, and clinical significance of a dyadic, web-based, psychosocial and physical activity self-management program (tempo) tailored to the needs of men with prostate cancer and their caregivers: a multi-center randomized pilot trial. Curr Oncol. (2022) 29(2):785–804. 10.3390/curroncol29020067 35200566 PMC8871005

[22] Chambers SK Occhipinti S Schover L Nielsen L Zajdlewicz L Clutton S A randomised controlled trial of a couples-based sexuality intervention for men with localised prostate cancer and their female partners. Psychooncology. (2015) 24(7):748–56. 10.1002/pon.3726 25483780

[23] Moher D Liberati A Tetzlaff J Altman DG. Preferred reporting items for systematic reviews and meta-analyses: the PRISMA statement. PLoS Med. (2009) 6(7):e1000097. 10.1371/journal.pmed.1000097 19621072 PMC2707599

[24] Higgins JPT Thomas J Chandler J Cumpston M Li T Page MJ Cochrane Handbook for Systematic Reviews of Interventions Version 6.4 (updated August 2023). Cochrane (2023). Available at: https://training.cochrane.org/handbook (Accessed November 08, 2024).

[25] Giesler RB Given B Given CW Rawl S Monahan P Burns D Improving the quality of life of patients with prostate carcinoma: a randomized trial testing the efficacy of a nurse-driven intervention. Cancer. (2005) 104(4):752–62. 10.1002/cncr.21231 15986401

[26] Gong R Heller A Moreno PI Yanez B Penedo FJ. Low social well-being in advanced and metastatic prostate cancer: effects of a randomized controlled trial of cognitive behavioral stress management. Int J Behav Med. (2024) 35(14):1–14. 10.1007/s12529-024-10270-w PMC11333730 38378974

[27] Langlais CS Chen YH Van Blarigan EL Kenfield SA Kessler ER Daniel K Quality of life of prostate cancer survivors participating in a remotely delivered web-based behavioral intervention pilot randomized trial. Integr Cancer Ther. (2022) 21:15347354211063500. 10.1177/15347354211063500 35389288 PMC9016550

[28] Tagai EK Miller SM Hudson SV Diefenbach MA Handorf E Bator A Improved cancer coping from a web-based intervention for prostate cancer survivors: a randomized controlled trial. Psychooncology. (2021) 30(9):1466–75. 10.1002/pon.5701 33855796 PMC9053312

[29] Wennerberg C Hellström A Schildmeijer K Ekstedt M. Effects of web-based and Mobile self-care support in addition to standard care in patients after radical prostatectomy: randomized controlled trial. Jmir Cancer. (2023) 9:e44320. 10.2196/44320 37672332 PMC10512115

[30] Heydenreich M Puta C Gabriel HHW Dietze A Wright P Zermann D. Does trunk muscle training with an oscillating rod improve urinary incontinence after radical prostatectomy? A prospective randomized controlled trial. Clin Rehabil. (2020) 34(3):320–33. 10.1177/0269215519893096 31858823 PMC7029439

[31] Aliperti L Patil D Mehta A Filson C Crociani C Sanda M Long-term health related quality of life in prostate cancer patients requiring radiotherapy after radical prostatectomy. J Urol. (2017) 197(4):e360. 10.1016/j.juro.2017.02.863

[32] Dronca R Rao R Maniaci M Odedina F Kaninjing E Ashing K Feasibility of patient-centered home care (PCHC) to reduce disparities in black men (BM) with advanced prostate cancer (CAP): an Iccare Consortium for Prostate Cancer in Black Men Project. Cancer Epidemiol Biomarkers Prev. (2023) 32(1):B046. 10.1158/1538-7755.DISP22-B046

[33] Xu S Guo P Fuller GP Dobias CA Idiagbonya E Song L. Neighborhood deprivation and living with prostate cancer: patients’ and Partners’ psychosocial behavioral Status, symptoms, and quality of life. Cancer Epidemiol Biomarkers Prev. (2022) 31(1 SUPPL):PO-176. 10.1158/1538-7755.DISP21-PO-176

[34] Evans HEL Galvão DA Forbes CC Girard D Vandelanotte C Newton RU Acceptability and preliminary efficacy of a web- and telephone-based personalised exercise intervention for individuals with metastatic prostate cancer: the exerciseguide pilot randomised controlled trial. Cancers (Basel). (2021) 13(23):5925. 10.3390/cancers13235925 34885036 PMC8656540

[35] Badger TA Segrin C Figueredo AJ Harrington J Sheppard K Passalacqua S Psychosocial interventions to improve quality of life in prostate cancer survivors and their intimate or family partners. Qual Life Res. (2011) 20(6):833–44. 10.1007/s11136-010-9822-2 21170682 PMC3117079

[36] Chambers SK Occhipinti S Foley E Clutton S Legg M Berry M Mindfulness-based cognitive therapy in advanced prostate cancer: a randomized controlled trial. J Clin Oncol. (2017) 35(3):291–7. 10.1200/JCO.2016.68.8788 27870567

[37] Goode PS Johnson TM 2nd Newman DK Vaughan CP Echt KV Markland AD Perioperative mobile telehealth program for post-prostatectomy incontinence: a randomized clinical trial. J Urol. (2022) 208(2):379–87. 10.1097/ju.0000000000002697 35389239

[38] Song L Guo P Tan X Chen RC Nielsen ME Birken SA Enhancing survivorship care planning for patients with localized prostate cancer using a couple-focused web-based, Mhealth program: the results of a pilot feasibility study. J Cancer Surviv. (2021) 15(1):99–108. 10.1007/s11764-020-00914-7 32681304 PMC7855003

[39] Wittmann D Mehta A Bober SL Zhu Z Daignault-Newton S Dunn RL Truenth sexual recovery intervention for couples coping with prostate cancer: randomized controlled trial results. Cancer. (2022) 128(7):1513–22. 10.1002/cncr.34076 34985771

[40] Wootten AC Meyer D Abbott JM Chisholm K Austin DW Klein B An online psychological intervention can improve the sexual satisfaction of men following treatment for localized prostate cancer: outcomes of a randomised controlled trial evaluating my road ahead. Psychooncology. (2017) 26(7):975–81. 10.1002/pon.4244 27503036

[41] Davis KM Dawson D Kelly S Red S Penek S Lynch J Monitoring of health-related quality of life and symptoms in prostate cancer survivors: a randomized trial. J Support Oncol. (2013) 11(4):174–82. 10.12788/j.suponc.0013 24645337

[42] Hunter M Stefanopoulou E Yousaf O Grunfeld E. A randomised controlled trial of a cognitive behavioural intervention for men who have hot flushes following prostate cancer treatment (Mancan). Psychooncology. (2014) 23:126–7. 10.1111/j.1099-1611.2014.3694

[43] Lambert SD McElduff P Girgis A Levesque JV Regan TW Turner J A pilot, multisite, randomized controlled trial of a self-directed coping skills training intervention for couples facing prostate cancer: accrual, retention, and data collection issues. Support Care Cancer. (2016) 24(2):711–22. 10.1007/s00520-015-2833-3 26184499

[44] Lange L Fink J Bleich C Graefen M Schulz H. Effectiveness, acceptance and satisfaction of guided chat groups in psychosocial aftercare for outpatients with prostate cancer after prostatectomy. Internet Interv Appl Inf Technol Mental Behav Health. (2017) 9:57–64. 10.1016/j.invent.2017.06.001 PMC6096258 30135838

[45] Saengryeol P Kwangnam K Hyun Kyu A Jong Won K Gyurang M Byung Ha C Impact of lifestyle intervention for patients with prostate cancer. Am J Health Behav. (2020) 44(1):90–9. 10.5993/AJHB.44.1.10 31783936

[46] McCaughan E Curran C Northouse L Parahoo K. Evaluating a psychosocial intervention for men with prostate cancer and their partners: outcomes and lessons learned from a randomized controlled trial. Appl Nurs Res. (2018) 40:143–51. 10.1016/j.apnr.2018.01.008 29579490

[47] Park YH Lee JI Lee JY Cheong IY Hwang JH Seo SI Internet of things-based lifestyle intervention for prostate cancer patients on androgen deprivation therapy: a prospective, multicenter, randomized trial. Am J Cancer Res. (2021) 11(11):5496–507. 34873475 PMC8640797

[48] Qan'ir Y Song L. Systematic review of technology-based interventions to improve anxiety, depression, and health-related quality of life among patients with prostate cancer. Psychooncology. (2019) 28(8):1601–13. 10.1002/pon.5158 31222956 PMC7465427

[49] James C Brunckhorst O Eymech O Stewart R Dasgupta P Ahmed K. Fear of cancer recurrence and PSA anxiety in patients with prostate cancer: a systematic review. Support Care Cancer. (2022) 30(7):5577–89. 10.1007/s00520-022-06876-z 35106656 PMC9135793

[50] Meissner VH Peter C Ankerst DP Schiele S Gschwend JE Herkommer K Prostate cancer-related anxiety among long-term survivors after radical prostatectomy: a longitudinal study. Cancer Med. (2023) 12(4):4842–51. 10.1002/cam4.5304 36254563 PMC9972104

[51] Watts S Leydon G Birch B Prescott P Lai L Eardley S Depression and anxiety in prostate cancer: a systematic review and meta-analysis of prevalence rates. BMJ Open. (2014) 4(3):e003901. 10.1136/bmjopen-2013-003901 24625637 PMC3963074

[52] Zhao X Sun M Yang Y. Effects of social support, hope and resilience on depressive symptoms within 18 months after diagnosis of prostate cancer. Health Qual Life Outcomes. (2021) 19(1):15. 10.1186/s12955-020-01660-1 33413485 PMC7792299

[53] Ilie G Rendon R Mason R MacDonald C Kucharczyk MJ Patil N A comprehensive 6-Mo prostate cancer patient empowerment program decreases psychological distress among men undergoing curative prostate cancer treatment: a randomized clinical trial. Eur Urol. (2023) 83(6):561–70. 10.1016/j.eururo.2023.02.009 36822969

[54] Occhipinti S Zajdlewicz L Coughlin GD Yaxley JW Dunglison N Gardiner RA A prospective study of psychological distress after prostate cancer surgery. Psychooncology. (2019) 28(12):2389–95. 10.1002/pon.5263 31659807

[55] Penedo FJ Oswald LB Kronenfeld JP Garcia SF Cella D Yanez B. The increasing value of Ehealth in the delivery of patient-centred cancer care. Lancet Oncol. (2020) 21(5):e240–e51. 10.1016/s1470-2045(20)30021-8 32359500 PMC7643123

[56] Tayama J Hamaguchi T Koizumi K Yamamura R Okubo R Kawahara JI Efficacy of an Ehealth self-management program in reducing irritable bowel syndrome symptom severity: a randomized controlled trial. Sci Rep. (2024) 14(1):4. 10.1038/s41598-023-50293-z 38172498 PMC10764726

[57] Knowles SR Mikocka-Walus A. Utilization and efficacy of internet-based Ehealth technology in gastroenterology: a systematic review. Scand J Gastroenterol. (2014) 49(4):387–408. 10.3109/00365521.2013.865259 24494974

[58] WHOQOL Group. Development of the World Health Organization WHOQOL-BREF quality of life assessment. Psychol Med. (1998) 28(3):551–8. 9626712 10.1017/s0033291798006667

[59] Buneviciene I Mekary RA Smith TR Onnela JP Bunevicius A. Can Mhealth interventions improve quality of life of cancer patients? A systematic review and meta-analysis. Crit Rev Oncol Hematol. (2021) 157:103123. 10.1016/j.critrevonc.2020.103123 33190065 PMC7574857

[60] Leach CR Hudson SV Diefenbach MA Wiseman KP Sanders A Coa K Cancer health self-efficacy improvement in a randomized controlled trial. Cancer. (2022) 128(3):597–605. 10.1002/cncr.33947 34668569 PMC9930867

[61] Jackson SR Yu P Armany D Occhipinti S Chambers S Leslie S Ehealth literacy in prostate cancer: a systematic review. Patient Educ Couns. (2024) 123:108193. 10.1016/j.pec.2024.108193 38354430

[62] Carter DD Robinson K Forbes J Hayes S. Experiences of mobile health in promoting physical activity: a qualitative systematic review and meta-ethnography. PLoS One. (2018) 13(12):e0208759. 10.1371/journal.pone.0208759 30557396 PMC6296673

[63] Peng S Wei Y Ye L Jin X Huang L. Application of mobile internet management in the continuing care of patients after radical prostatectomy. Sci Rep. (2024) 14(1):31520. 10.1038/s41598-024-83303-9 39733026 PMC11682269

[64] Porter LS Keefe FJ Garst J Baucom DH McBride CM McKee DC Caregiver-assisted coping skills training for lung cancer: results of a randomized clinical trial. J Pain Symptom Manage. (2011) 41(1):1–13. 10.1016/j.jpainsymman.2010.04.014 20832982 PMC3010525

