# Supplementary material for: Effectiveness of mobile health in symptom management of prostate cancer patients: a systematic review and meta-analysis
Source: Front Digit Health. 2025 May 7;7:1584764. doi: 10.3389/fdgth.2025.1584764 (PMC12092381; doi:10.3389/fdgth.2025.1584764)
Supplement: Supplementary file 1 [file Datasheet1.pdf]

S1. Search strategy

Database	#	Search strategy	Results
PubMed	1	"Prostatic Neoplasms"[MeSH Terms]	155,982
	2	"Prostate"[MeSH Terms] OR "prostat*"[Title/Abstract]	272,682
	3	"neoplasm*"[Title/Abstract] OR "cancer*"[Title/Abstract] OR "carcinoma*"[Title/Abstract] OR "neoplasia*"[Title/Abstract] OR "tumor*"[Title/Abstract] OR "tumour*"[Title/Abstract] OR "malignan*"[Title/Abstract]	4,274,148
	4	#2 AND #3	203,734
	5	#1 OR #4	224,260
	6	(("Internet"[Mesh]) OR "Smartphone"[Mesh]) OR "Telecommunications"[Mesh]	227,857
	7	camera*[Title/Abstract] OR phone*[Title/Abstract] OR Smartphone[Title/Abstract] OR teleconferenc*[Title/Abstract] OR telephone*[Title/Abstract] OR telepsychiatry[Title/Abstract] OR telemedicine*[Title/Abstract] OR video*[Title/Abstract] OR webcam*[Title/Abstract] OR mobile tablet[Title/Abstract] OR telehealth*[Title/Abstract] OR telecommunications*[Title/Abstract] OR telecasting[Title/Abstract] OR e-health[Title/Abstract] OR e-medicine[Title/Abstract] OR mobile health[Title/Abstract] OR information technology[Title/Abstract] OR information communication technology[Title/Abstract] OR internet[Title/Abstract] OR web-based[Title/Abstract] OR computer[Title/Abstract] OR Software[Title/Abstract] OR videoconferencing[Title/Abstract] OR remote sensing technology[Title/Abstract]	1,019,331
	8	remote*[Title/Abstract] AND (test*[Title/Abstract] OR diagnos*[Title/Abstract] OR consult*[Title/Abstract] OR deliver*[Title/Abstract])	39,754
	9	#6 OR #7 OR #8	1,153,913
	10	("randomized controlled trial"[Publication Type] OR "controlled clinical trial"[Publication Type] OR "randomized"[Title/Abstract] OR "placebo"[Title/Abstract] OR "clinical trials as topic"[MeSH Terms:noexp] OR "randomly"[Title/Abstract] OR "trial"[Title]) NOT ("animals"[MeSH Terms] NOT ("humans"[MeSH Terms] AND "animals"[MeSH Terms]))	1,524,470
	11	#5 AND #9 AND #10	778
Cochrane library	1	MeSH descriptor: [Prostatic Neoplasms] explode all trees	9007
	2	MeSH descriptor: [Prostatism] explode all trees	92
	3	(prostat*):ti,ab,kw	28359
	4	#2 OR #3	28359
	5	(Neoplasm* OR cancer* OR carcinoma* OR neoplasia* OR tumor* OR tumour* OR	287512

		malignan*):ti,ab,kw	
	6	#4 AND #5	19724
	7	#1 OR #6	19724
	8	MeSH descriptor: [Internet] explode all trees	6749
	9	MeSH descriptor: [Smartphone] explode all trees	1224
	10	MeSH descriptor: [Telecommunications] explode all trees	11933
	11	#8 OR #9 OR #10	17499
	12	(camera* OR phone* OR Smartphone OR teleconferenc* OR telephone* OR telepsychiatry OR telemedicine* OR video* OR webcam* OR mobile tablet OR telehealth* OR telecommunications* OR telecasting OR e-health OR e-medicine OR mobile health OR information technology OR information communication technology OR internet OR web-based OR computer OR Software OR videoconferencing OR remote sensing technology):ti,ab,kw	191012
	13	(remote* AND (test* or diagnos* or consult* or deliver*)):ti,ab,kw	7561
	14	#11 OR #12 OR #13	195762
	15	#7 AND #14	1812
EMBASE	1	'prostate tumor'/exp	321557
	2	'prostate'/exp OR prostat*:ab,ti	400903
	3	neoplasm*:ab,ti OR cancer*:ab,ti OR carcinoma*:ab,ti OR neoplasia*:ab,ti OR tumor*:ab,ti OR tumour*:ab,ti OR malignan*:ab,ti	5785107
	4	#2 AND #3	304070
	5	#1 OR #4	372828
	6	'internet'/exp OR 'smartphone'/exp OR 'telecommunications'/exp	284378
	7	camera*:ab,ti OR phone*:ab,ti OR smartphone:ab,ti OR teleconferenc*:ab,ti OR telephone*:ab,ti OR telepsychiatry:ab,ti OR telemedicine*:ab,ti OR video*:ab,ti OR webcam*:ab,ti OR 'mobile tablet':ab,ti OR telehealth*:ab,ti OR telecommunications*:ab,ti OR telecasting:ab,ti OR 'e health':ab,ti OR 'e medicine':ab,ti OR 'mobile health':ab,ti OR 'information technology':ab,ti OR 'information communication technology':ab,ti OR internet:ab,ti OR 'web based':ab,ti OR computer:ab,ti OR software:ab,ti OR videoconferencing:ab,ti OR 'remote sensing technology':ab,ti	1381542
	8	remote*:ab,ti AND (test*:ab,ti OR diagnos*:ab,ti OR consult*:ab,ti OR deliver*:ab,ti)	55151
	9	#6 OR #7 OR #8	1534053
	10	'crossover procedure':de OR 'double-blind procedure':de OR 'randomized controlled trial':de OR 'single-blind procedure':de OR (random* OR factorial* OR crossover* OR cross NEXT/1 over* OR placebo* OR doubl* NEAR/1 blind* OR singl* NEAR/1 blind* OR assign* OR allocat* OR volunteer*):de,ab,ti	1740647
	11	#5 AND #9 AND #10	790
Web of Science	1	TS=prostatic neoplasms	8189
	2	TS=prostate OR TS=prostat*	357072
	3	TS=Neoplasm* OR TS=cancer* OR TS=carcinoma* OR TS=neoplasia* OR TS=tumor*[TS] OR TS=tumour* OR TS=malignan*	3930509
	4	#2 AND #3	287072

5	#1 OR #4	287072
6	TS=(Internet OR Smartphone OR Telecommunications)	489562
7	TS=(camera* OR phone* OR Smartphone OR teleconferenc* OR telephone* OR telepsychiatry OR telemedicine* OR video* OR webcam* OR mobile tablet OR telehealth* OR telecommunications* OR telecasting OR e-health OR e-medicine OR mobile health OR information technology OR information communication technology OR internet OR web-based OR computer OR Software OR videoconferencing OR remote sensing technology)	3030170
8	TS=(remote* AND (test* or diagnos* or consult* or deliver*))	93640
9	#6 OR #7 OR #8	3109743
10	TS= clinical trial* OR TS=research design OR TS=comparative stud* OR TS=evaluation stud* OR TS=controlled trial* OR TS=follow-up stud* OR TS=prospective stud* OR TS=random* OR TS=placebo* OR TS=(single blind*) OR TS=(double blind*)	6607525
11	#5 AND #9 AND #10	2910

Search date: 2024/11/8
